# Dataset on solar contributions by thermal solar systems in Chile applying Chilean and Spanish regulations

**DOI:** 10.1016/j.dib.2019.104505

**Published:** 2019-09-16

**Authors:** Luis M. López-Ochoa, Konstantin Verichev, Jesús Las-Heras-Casas, Manuel Carpio

**Affiliations:** a TENECO Research Group, Department of Mechanical Engineering, University of La Rioja, Calle San José de Calasanz, 31, Logroño, La Rioja, Spain; b Institute of Civil Engineering, Faculty of Engineering Sciences, Universidad Austral de Chile, Avenida General Lagos, 2050, Valdivia, Chile; c Department of Construction Engineering and Management, School of Engineering, Pontificia Universidad Católica de Chile, Avenida Vicuña Mackenna, 4860, Santiago, Chile; d UC Energy Research Center, Pontificia Universidad Católica de Chile, Avenida Vicuña Mackenna, 4860, Santiago, Chile

**Keywords:** Solar contribution, Thermal solar system, Solar domestic hot water regulation, Chile, Residential sector, EPBD

## Abstract

The data presented in this article are related to the research article entitled “Solar domestic hot water regulation in the Latin American residential sector with the implementation of the Energy Performance of Buildings Directive: The case of Chile” (López-Ochoa et al., 2019), which evaluates the possibility of adapting Spanish solar domestic hot water regulations in Chile, with the objective of presenting the potential impact of the Energy Performance of Buildings Directive in Latin America. This dataset was made publicly available to show the possible energy savings with the thermal solar systems proposed to enable the use of these data by other researchers as well as designers, installers and decision-makers.

**Table:** Specifications Table

Subject area	Energy and Buildings
More specific subject area	Solar domestic hot water for multifamily buildings
Type of data	Tables
How data was acquired	f-Chart method
Data format	Raw
Experimental factors	Evaluation of the solar contribution of thermal solar systems, applying Chilean and Spanish regulations transposed from the Energy Performance of Buildings Directive to a multifamily building
Experimental features	Determination of the regulation with which the best use of solar thermal energy is obtained
Data source location	Main continental communes of Chile
Data accessibility	Data are available with this article
Related research article	Luis M. López-Ochoa, Konstantin Verichev, Jesús Las-Heras-Casas, Manuel Carpio Solar domestic hot water regulation in the Latin American residential sector with the implementation of the Energy Performance of Buildings Directive: The case of Chile Energy DOI: 10.1016/j.energy.2019.115985

**Table:** 

**Value of the data** • Using the data presented in this article, researchers can evaluate the possibility of improving the legislation on solar domestic hot water in Latin American countries by adapting the European Energy Performance of Buildings Directive. • The data show the main characteristics of the thermal solar systems to be installed in the main continental communes of Chile to comply with the Chilean regulations and the transposed Spanish regulations of the Energy Performance of Buildings Directives 2002 and 2010. • The data reveal the energy savings that can be obtained with the different proposed thermal solar systems, which can be evaluated at the energy, environmental and economic levels in future research. • These data can serve as a benchmark for designers and installers of thermal solar systems in Chile and can suggest systems that can take better advantage of solar thermal energy. • Other researchers and decision-makers can use the data of this work to improve the Latin American regulations in this matter.

## 1 Data

The data presented in this article are related to [1]. These data present the main characteristics of the Chilean continental communes and the thermal solar systems used in each commune to comply with Chilean regulation NCL [2], [3], [4] and Spanish regulations CTE 2009 [5], [6], [7] (transposed from the Energy Performance of Buildings Directive 2002 [8]) and CTE 2013 [9], [10], [11] (transposed from the Energy Performance of Buildings Directive 2010 [12]), which are reported in Table 1. In addition, the results obtained via the thermal solar systems in each commune are presented by solar climate zone, with application of the different legislations and the f-chart verification algorithm of the Chilean Ministry of Energy [13], and are reported in Table 2, Table 3, Table 4, Table 5, Table 6, Table 7.

**Table 1:** Main characteristics of communes and thermal solar systems employed, as well as the annual solar contribution and the annual minimum solar contribution, according to regulations, by solar climate zone.

SCZ	Commune	R	L	I	NCL [2], [3], [4]				CTE 2009 [5], [6], [7]				CTE 2013 [9], [10], [11]			
					N	V/A	f	f_min_	N	V/A	f	f_min_	N	V/A	f	f_min_
A	Antofagasta	2360	−24.27	25.00	8	168.16	0.6394	0.6375	10	134.53	0.7132	0.7000	9	174.39	0.6279	0.6000
	Arica	2342	−18.54	20.00	8	168.16	0.6970	0.6375	9	149.48	0.7208	0.7000	9	174.39	0.6853	0.6000
	Calama	2506	−22.16	20.00	8	168.16	0.6936	0.6375	9	149.48	0.7181	0.7000	9	174.39	0.6815	0.6000
	Chañaral	2055	−26.37	25.00	9	149.48	0.6879	0.6375	10	134.53	0.7044	0.7000	9	174.39	0.6243	0.6000
	Copiapó	2174	−27.32	25.00	10	134.53	0.6799	0.6375	12	112.11	0.7340	0.7000	10	156.95	0.6144	0.6000
	Illapel	1979	−31.55	30.00	10	134.53	0.6704	0.6375	12	112.11	0.7201	0.7000	10	156.95	0.6088	0.6000
	Iquique	2067	−20.94	20.00	9	149.48	0.6850	0.6375	10	134.53	0.7016	0.7000	9	174.39	0.6213	0.6000
	Pozo Almonte	2264	−20.77	20.00	8	168.16	0.6711	0.6375	10	134.53	0.7443	0.7000	9	174.39	0.6596	0.6000
	Putre	2395	−18.43	20.00	9	149.48	0.6660	0.6375	11	122.30	0.7305	0.7000	10	156.95	0.6482	0.6000
	Tocopilla	2204	−22.00	20.00	8	168.16	0.6717	0.6375	10	134.53	0.7435	0.7000	9	174.39	0.6603	0.6000
	Vallenar	2088	−28.59	30.00	9	149.48	0.6517	0.6375	11	122.30	0.7127	0.7000	10	156.95	0.6349	0.6000
B	Colina	1843	−33.13	35.00	9	149.48	0.5847	0.5610	13	103.48	0.7106	0.7000	11	142.68	0.6088	0.6000
	La Serena	1814	−29.79	30.00	9	149.48	0.5836	0.5610	11	122.30	0.6400	0.6000	9	174.39	0.5254	0.5000
	Los Andes	1706	−32.95	35.00	12	112.11	0.5951	0.5610	13	103.48	0.6002	0.6000	11	142.68	0.5017	0.5000
	Ovalle	1749	−30.67	30.00	9	149.48	0.5694	0.5610	11	122.30	0.6244	0.6000	9	174.39	0.5125	0.5000
	Puente Alto	1866	−33.59	35.00	9	149.48	0.5999	0.5610	13	103.48	0.7263	0.7000	11	142.68	0.6242	0.6000
	San Bernardo	1792	−33.63	35.00	9	149.48	0.5770	0.5610	11	122.30	0.6311	0.6000	9	174.39	0.5207	0.5000
	San Felipe	1929	−32.74	35.00	8	168.16	0.5714	0.5610	12	112.11	0.7153	0.7000	10	156.95	0.6050	0.6000
	Santiago	1843	−33.45	35.00	9	149.48	0.5891	0.5610	13	103.48	0.7140	0.7000	11	142.68	0.6131	0.6000
	Talagante	1776	−33.68	35.00	9	149.48	0.5726	0.5610	11	122.30	0.6264	0.6000	9	174.39	0.5166	0.5000
C	Angol	1483	−37.77	40.00	10	134.53	0.4975	0.4845	8	168.16	0.3974	0.3000	9	174.39	0.4113	0.3000
	Cauquenes	1567	−35.97	35.00	10	134.53	0.5337	0.4845	10	134.53	0.5079	0.5000	9	174.39	0.4440	0.4000
	Chillán	1534	−36.62	35.00	9	149.48	0.4922	0.4845	10	134.53	0.5056	0.5000	9	174.39	0.4420	0.4000
	Concepción	1497	−36.83	35.00	10	134.53	0.5173	0.4845	8	168.16	0.4156	0.3000	9	174.39	0.4299	0.3000
	Coquimbo	1701	−30.23	30.00	8	168.16	0.5094	0.4845	11	122.30	0.6102	0.6000	10	156.95	0.5410	0.5000
	Curicó	1615	−35.20	35.00	9	149.48	0.5066	0.4845	10	134.53	0.5203	0.5000	9	174.39	0.4553	0.4000
	La Ligua	1543	−32.35	30.00	9	149.48	0.5213	0.4845	10	134.53	0.5346	0.5000	9	174.39	0.4699	0.4000
	Linares	1563	−35.96	35.00	9	149.48	0.4987	0.4845	10	134.53	0.5120	0.5000	9	174.39	0.4484	0.4000
	Los Ángeles	1498	−37.41	35.00	10	134.53	0.5150	0.4845	8	168.16	0.4135	0.3000	9	174.39	0.4278	0.3000
	Melipilla	1580	−33.74	35.00	8	168.16	0.4937	0.4845	9	149.48	0.5115	0.5000	9	174.39	0.4848	0.4000
	Pichilemu	1546	−34.38	35.00	9	149.48	0.5153	0.4845	10	134.53	0.5285	0.5000	9	174.39	0.4646	0.4000
	Quillota	1499	−32.90	35.00	9	149.48	0.5128	0.4845	8	168.16	0.4469	0.3000	9	174.39	0.4620	0.3000
	Quilpué	1617	−33.15	35.00	8	168.16	0.4881	0.4845	9	149.48	0.5061	0.5000	9	174.39	0.4792	0.4000
	Rancagua	1602	−34.13	35.00	9	149.48	0.5187	0.4845	10	134.53	0.5323	0.5000	9	174.39	0.4669	0.4000
	San Antonio	1458	−33.67	35.00	9	149.48	0.5000	0.4845	8	168.16	0.4355	0.3000	9	174.39	0.4504	0.3000
	San Fernando	1657	−34.74	35.00	9	149.48	0.5136	0.4845	10	134.53	0.5277	0.5000	9	174.39	0.4613	0.4000
	Talca	1509	−35.43	35.00	10	134.53	0.5222	0.4845	8	168.16	0.4210	0.3000	9	174.39	0.4354	0.3000
D	Chile Chico	1284	−46.78	45.00	9	149.48	0.4097	0.4080	8	168.16	0.3528	0.3000	9	174.39	0.3654	0.3000
	Coyhaique	1347	−45.55	45.00	9	149.48	0.4311	0.4080	8	168.16	0.3718	0.3000	9	174.39	0.3849	0.3000
	La Unión	1308	−40.20	40.00	9	149.48	0.4180	0.4080	8	168.16	0.3608	0.3000	9	174.39	0.3737	0.3000
	Lebu	1401	−37.68	40.00	8	168.16	0.4113	0.4080	8	168.16	0.3897	0.3000	9	174.39	0.4034	0.3000
	Osorno	1271	−40.61	40.00	9	149.48	0.4115	0.4080	8	168.16	0.3552	0.3000	9	174.39	0.3679	0.3000
	Puerto Montt	1212	−41.49	40.00	10	134.53	0.4176	0.4080	8	168.16	0.3318	0.3000	9	174.39	0.3438	0.3000
	Temuco	1405	−38.67	40.00	8	168.16	0.4137	0.4080	8	168.16	0.3920	0.3000	9	174.39	0.4057	0.3000
	Valdivia	1338	−39.82	40.00	9	149.48	0.4338	0.4080	8	168.16	0.3751	0.3000	9	174.39	0.3883	0.3000
	Valparaíso	1431	−33.13	35.00	8	168.16	0.4452	0.4080	8	168.16	0.4225	0.3000	9	174.39	0.4370	0.3000
E	Aysén	1126	−45.98	45.00	8	168.16	0.3423	0.3315	8	168.16	0.3238	0.3000	9	174.39	0.3356	0.3000
	Castro	1163	−42.47	40.00	8	168.16	0.3399	0.3315	8	168.16	0.3215	0.3000	9	174.39	0.3332	0.3000
	Chaitén	1188	−43.09	45.00	8	168.16	0.3430	0.3315	8	168.16	0.3244	0.3000	9	174.39	0.3362	0.3000
	Cochrane	1193	−47.36	45.00	8	168.16	0.3484	0.3315	8	168.16	0.3295	0.3000	9	174.39	0.3414	0.3000
	Porvenir	1026	−53.32	55.00	10	134.53	0.3564	0.3315	9	149.48	0.3099	0.3000	10	156.95	0.3179	0.3000
F	Natales	922	−50.64	50.00	8	168.16	0.2727	0.2550	10	134.53	0.3096	0.3000	11	142.68	0.3152	0.3000
	Punta Arenas	872	−53.65	55.00	8	168.16	0.2554	0.2550	11	122.30	0.3127	0.3000	12	130.79	0.3162	0.3000

SCZ: solar climate zone; R: mean annual global solar radiation on horizontal surface, in kWh/m^2^·year; L: latitude, in °; I: angle of inclination of the solar collector, in °; N: number of solar collectors; V/A: relationship between the accumulator volume and the solar collector area used, in l/m^2^; f: annual solar contribution; f_min_: annual minimum solar contribution.

**Table 2:** Domestic hot water energy demand (ED), in kWh; domestic hot water energy demand met by the thermal solar system (TSS ED), in kWh; and solar contribution (SC) according to commune, regulation and month in solar climate zone A.

Commune	Regulation	Parameter	Jan	Feb	Mar	Apr	May	Jun	Jul	Aug	Sep	Oct	Nov	Dec
Antofagasta	NCL [2], [3], [4]	ED	3037	2814	3115	3137	3378	3354	3485	3378	3194	3154	3005	3057
		TSS ED	2097	2032	2158	1917	1911	1816	1907	1945	1915	2173	2164	2330
		SC	0.6906	0.7221	0.6927	0.6112	0.5657	0.5414	0.5472	0.5757	0.5996	0.6891	0.7202	0.7623
	CTE 2009 [5], [6], [7]	ED	3240	3001	3323	3346	3603	3577	3717	3603	3407	3364	3206	3261
		TSS ED	2486	2405	2559	2287	2287	2177	2287	2326	2287	2579	2562	2749
		SC	0.7675	0.8012	0.7701	0.6835	0.6348	0.6086	0.6151	0.6456	0.6712	0.7665	0.7991	0.8431
	CTE 2013 [9], [10], [11]	ED	3505	3247	3595	3620	3898	3870	4022	3898	3685	3640	3468	3527
		TSS ED	2378	2304	2446	2172	2163	2055	2159	2202	2169	2464	2454	2644
		SC	0.6785	0.7096	0.6806	0.6000	0.5550	0.5310	0.5368	0.5649	0.5885	0.6770	0.7077	0.7495
Arica	NCL [2], [3], [4]	ED	2628	2374	2628	2610	2804	2779	2911	2862	2751	2765	2628	2706
		TSS ED	1897	1763	1964	1864	1839	1713	1827	1865	1911	2059	1970	1944
		SC	0.7216	0.7427	0.7474	0.7142	0.6559	0.6162	0.6279	0.6518	0.6945	0.7447	0.7497	0.7183
	CTE 2009 [5], [6], [7]	ED	2804	2532	2804	2784	2991	2964	3105	3053	2934	2949	2804	2887
		TSS ED	2090	1942	2164	2055	2030	1892	2019	2060	2108	2269	2171	2143
		SC	0.7456	0.7670	0.7719	0.7382	0.6789	0.6384	0.6504	0.6748	0.7185	0.7695	0.7744	0.7424
	CTE 2013 [9], [10], [11]	ED	3033	2739	3033	3011	3235	3207	3359	3303	3174	3190	3033	3123
		TSS ED	2152	2001	2230	2115	2085	1941	2071	2115	2167	2337	2236	2206
		SC	0.7097	0.7305	0.7352	0.7022	0.6445	0.6053	0.6167	0.6404	0.6827	0.7324	0.7374	0.7063
Calama	NCL [2], [3], [4]	ED	2969	2708	2998	2949	3174	3156	3261	3154	2986	2989	2864	2950
		TSS ED	2048	1999	2152	1984	2023	1941	1997	2025	1996	2271	2254	2390
		SC	0.6896	0.7381	0.7176	0.6727	0.6374	0.6150	0.6123	0.6420	0.6684	0.7598	0.7869	0.8102
	CTE 2009 [5], [6], [7]	ED	3167	2889	3198	3145	3385	3366	3479	3364	3185	3188	3055	3146
		TSS ED	2261	2206	2375	2192	2237	2148	2210	2239	2206	2504	2484	2632
		SC	0.7140	0.7635	0.7426	0.6968	0.6609	0.6381	0.6353	0.6655	0.6925	0.7856	0.8131	0.8367
	CTE 2013 [9], [10], [11]	ED	3426	3125	3460	3403	3662	3642	3763	3640	3446	3449	3305	3404
		TSS ED	2322	2268	2440	2249	2292	2199	2262	2294	2263	2576	2558	2713
		SC	0.6776	0.7256	0.7053	0.6609	0.6259	0.6037	0.6011	0.6304	0.6566	0.7471	0.7740	0.7971
Chañaral	NCL [2], [3], [4]	ED	2473	2330	2580	2638	2881	2883	2998	2881	2694	2638	2487	2521
		TSS ED	2032	1870	1935	1657	1614	1478	1632	1780	1878	2054	1972	2113
		SC	0.8220	0.8024	0.7499	0.6281	0.5602	0.5125	0.5442	0.6178	0.6972	0.7788	0.7929	0.8382
	CTE 2009 [5], [6], [7]	ED	2637	2485	2752	2814	3074	3075	3198	3074	2874	2814	2653	2689
		TSS ED	2215	2039	2112	1812	1767	1619	1787	1948	2053	2242	2151	2303
		SC	0.8398	0.8204	0.7674	0.6440	0.5750	0.5264	0.5589	0.6339	0.7144	0.7967	0.8108	0.8563
	CTE 2013 [9], [10], [11]	ED	2853	2689	2977	3044	3325	3327	3460	3325	3109	3044	2870	2909
		TSS ED	2145	1969	2031	1727	1676	1530	1692	1853	1964	2160	2076	2232
		SC	0.7519	0.7324	0.6824	0.5674	0.5040	0.4600	0.4890	0.5573	0.6317	0.7095	0.7234	0.7672
Copiapó	NCL [2], [3], [4]	ED	3164	2954	3271	3316	3592	3589	3719	3602	3373	3329	3147	3193
		TSS ED	2497	2353	2418	2109	2028	1892	2018	2127	2225	2512	2482	2704
		SC	0.7892	0.7965	0.7393	0.6360	0.5645	0.5272	0.5427	0.5907	0.6596	0.7546	0.7889	0.8468
	CTE 2009 [5], [6], [7]	ED	3375	3151	3489	3537	3832	3829	3967	3842	3597	3551	3356	3406
		TSS ED	2859	2695	2778	2437	2352	2199	2344	2465	2569	2885	2844	3086
		SC	0.8471	0.8551	0.7962	0.6889	0.6138	0.5744	0.5910	0.6416	0.7140	0.8124	0.8473	0.9060
	CTE 2013 [9], [10], [11]	ED	3651	3409	3774	3827	4145	4142	4291	4156	3892	3842	3631	3685
		TSS ED	2619	2468	2528	2191	2098	1954	2085	2204	2313	2628	2603	2846
		SC	0.7175	0.7239	0.6698	0.5726	0.5061	0.4717	0.4859	0.5302	0.5944	0.6840	0.7169	0.7725
Illapel	NCL [2], [3], [4]	ED	2658	2567	2843	3005	3300	3344	3475	3349	3099	2969	2732	2706
		TSS ED	2419	2245	2208	1946	1725	1407	1581	1710	1922	2232	2308	2463
		SC	0.9102	0.8743	0.7768	0.6475	0.5227	0.4207	0.4548	0.5106	0.6202	0.7519	0.8448	0.9103
	CTE 2009 [5], [6], [7]	ED	2835	2739	3032	3206	3520	3567	3707	3572	3306	3167	2914	2887
		TSS ED	2736	2549	2522	2241	1998	1636	1837	1982	2218	2556	2626	2788
		SC	0.9652	0.9308	0.8319	0.6991	0.5677	0.4586	0.4956	0.5550	0.6709	0.8070	0.9009	0.9657
	CTE 2013 [9], [10], [11]	ED	3067	2963	3280	3468	3808	3859	4010	3864	3577	3426	3153	3123
		TSS ED	2570	2375	2322	2028	1786	1450	1631	1769	2000	2341	2437	2616
		SC	0.8379	0.8016	0.7078	0.5848	0.4690	0.3758	0.4067	0.4577	0.5591	0.6834	0.7731	0.8377
Iquique	NCL [2], [3], [4]	ED	2521	2321	2570	2581	2735	2732	2823	2813	2723	2765	2591	2580
		TSS ED	1942	1835	2021	1726	1699	1582	1558	1607	1745	2010	1997	2032
		SC	0.7702	0.7905	0.7863	0.6686	0.6210	0.5792	0.5521	0.5711	0.6410	0.7271	0.7709	0.7875
	CTE 2009 [5], [6], [7]	ED	2689	2476	2741	2753	2918	2914	3011	3001	2904	2949	2763	2752
		TSS ED	2118	2001	2204	1886	1858	1732	1706	1759	1909	2196	2180	2216
		SC	0.7877	0.8083	0.8040	0.6851	0.6367	0.5943	0.5666	0.5860	0.6573	0.7447	0.7888	0.8054
	CTE 2013 [9], [10], [11]	ED	2909	2679	2966	2979	3157	3153	3258	3246	3142	3190	2990	2977
		TSS ED	2042	1932	2127	1803	1770	1645	1618	1670	1819	2105	2098	2138
		SC	0.7020	0.7211	0.7171	0.6054	0.5607	0.5217	0.4967	0.5144	0.5790	0.6599	0.7019	0.7183
Pozo Almonte	NCL [2], [3], [4]	ED	2648	2436	2697	2694	2891	2864	2969	2920	2789	2813	2675	2726
		TSS ED	1870	1772	1927	1749	1757	1688	1736	1785	1819	2069	2015	2041
		SC	0.7062	0.7274	0.7145	0.6493	0.6077	0.5895	0.5847	0.6114	0.6525	0.7354	0.7532	0.7488
	CTE 2009 [5], [6], [7]	ED	2824	2598	2876	2874	3084	3055	3167	3115	2974	3001	2854	2907
		TSS ED	2205	2087	2272	2073	2089	2010	2068	2123	2157	2439	2371	2402
		SC	0.7809	0.8034	0.7899	0.7213	0.6774	0.6581	0.6530	0.6815	0.7253	0.8127	0.8309	0.8260
	CTE 2013 [9], [10], [11]	ED	3055	2811	3112	3109	3336	3305	3426	3370	3218	3246	3087	3145
		TSS ED	2122	2010	2186	1983	1991	1913	1967	2023	2063	2348	2287	2316
		SC	0.6944	0.7153	0.7026	0.6379	0.5968	0.5788	0.5741	0.6004	0.6410	0.7232	0.7408	0.7365
Putre	NCL [2], [3], [4]	ED	3368	3042	3368	3307	3514	3505	3612	3543	3354	3378	3222	3388
		TSS ED	2019	1899	2096	2208	2369	2154	2299	2341	2365	2541	2526	2219
		SC	0.5993	0.6242	0.6224	0.6679	0.6742	0.6147	0.6366	0.6607	0.7053	0.7523	0.7841	0.6550
	CTE 2009 [5], [6], [7]	ED	3593	3245	3593	3527	3749	3738	3852	3780	3577	3603	3437	3614
		TSS ED	2370	2226	2458	2584	2773	2531	2698	2743	2763	2959	2935	2598
		SC	0.6596	0.6861	0.6842	0.7327	0.7398	0.6770	0.7004	0.7257	0.7724	0.8212	0.8539	0.7189
	CTE 2013 [9], [10], [11]	ED	3887	3511	3887	3816	4055	4044	4168	4089	3870	3898	3718	3909
		TSS ED	2266	2132	2353	2481	2661	2417	2581	2629	2658	2858	2843	2492
		SC	0.5829	0.6073	0.6055	0.6501	0.6562	0.5977	0.6193	0.6429	0.6868	0.7332	0.7646	0.6375
Tocopilla	NCL [2], [3], [4]	ED	2521	2330	2580	2581	2774	2760	2862	2804	2675	2697	2544	2570
		TSS ED	1902	1788	1944	1693	1646	1522	1576	1623	1690	1966	1942	2000
		SC	0.7542	0.7675	0.7535	0.6560	0.5931	0.5513	0.5506	0.5790	0.6315	0.7292	0.7635	0.7782
	CTE 2009 [5], [6], [7]	ED	2689	2485	2752	2753	2959	2944	3053	2991	2854	2876	2713	2741
		TSS ED	2232	2098	2283	2003	1956	1814	1879	1931	2004	2316	2280	2344
		SC	0.8299	0.8441	0.8296	0.7276	0.6609	0.6160	0.6153	0.6458	0.7021	0.8051	0.8403	0.8551
	CTE 2013 [9], [10], [11]	ED	2909	2689	2977	2979	3202	3185	3303	3235	3087	3112	2935	2966
		TSS ED	2159	2030	2207	1920	1865	1724	1785	1839	1916	2231	2205	2271
		SC	0.7421	0.7552	0.7413	0.6447	0.5825	0.5412	0.5406	0.5686	0.6204	0.7171	0.7512	0.7658
Vallenar	NCL [2], [3], [4]	ED	2872	2682	2969	3024	3261	3278	3397	3290	3109	3066	2883	2920
		TSS ED	2203	2042	2141	1880	1733	1550	1749	1903	2036	2276	2170	2271
		SC	0.7673	0.7613	0.7212	0.6216	0.5314	0.4727	0.5149	0.5782	0.6549	0.7423	0.7526	0.7777
	CTE 2009 [5], [6], [7]	ED	3063	2861	3167	3226	3479	3497	3624	3510	3316	3271	3075	3115
		TSS ED	2553	2368	2489	2199	2037	1827	2060	2233	2379	2645	2518	2630
		SC	0.8333	0.8277	0.7860	0.6818	0.5857	0.5226	0.5684	0.6362	0.7175	0.8086	0.8188	0.8444
	CTE 2013 [9], [10], [11]	ED	3314	3095	3426	3490	3763	3783	3920	3797	3587	3539	3327	3370
		TSS ED	2481	2298	2409	2111	1944	1737	1962	2135	2287	2561	2442	2558
		SC	0.7487	0.7427	0.7031	0.6051	0.5166	0.4591	0.5003	0.5624	0.6376	0.7239	0.7341	0.7590

**Table 3:** Domestic hot water energy demand (ED), in kWh; domestic hot water energy demand met by the thermal solar system (TSS ED), in kWh; and solar contribution (SC) according to commune, regulation and month in solar climate zone B.

Commune	Regulation	Parameter	Jan	Feb	Mar	Apr	May	Jun	Jul	Aug	Sep	Oct	Nov	Dec
Colina	NCL [2], [3], [4]	ED	2697	2576	2852	3005	3271	3288	3427	3320	3118	3037	2770	2852
		TSS ED	2194	2011	2034	1679	1379	1107	1309	1488	1697	1998	2073	2205
		SC	0.8136	0.7805	0.7131	0.5586	0.4217	0.3366	0.3819	0.4483	0.5443	0.6577	0.7484	0.7729
	CTE 2009 [5], [6], [7]	ED	2876	2748	3042	3206	3489	3507	3655	3541	3326	3240	2954	3042
		TSS ED	2759	2550	2607	2204	1840	1487	1755	1982	2236	2588	2643	2799
		SC	0.9594	0.9281	0.8567	0.6876	0.5273	0.4240	0.4801	0.5598	0.6722	0.7988	0.8945	0.9201
	CTE 2013 [9], [10], [11]	ED	3112	2973	3291	3468	3774	3794	3954	3831	3598	3505	3196	3291
		TSS ED	2623	2407	2439	2021	1665	1338	1582	1796	2045	2400	2483	2640
		SC	0.8429	0.8097	0.7410	0.5829	0.4412	0.3527	0.4000	0.4690	0.5683	0.6847	0.7770	0.8019
La Serena	NCL [2], [3], [4]	ED	2843	2638	2920	2958	3174	3118	3271	3222	3052	3066	2892	2891
		TSS ED	1997	1841	1910	1637	1480	1303	1480	1619	1766	2031	1938	2036
		SC	0.7024	0.6978	0.6540	0.5532	0.4663	0.4179	0.4526	0.5025	0.5785	0.6622	0.6702	0.7043
	CTE 2009 [5], [6], [7]	ED	3032	2814	3115	3155	3385	3326	3489	3437	3256	3271	3085	3084
		TSS ED	2321	2142	2228	1919	1743	1537	1745	1905	2070	2370	2260	2368
		SC	0.7655	0.7612	0.7151	0.6083	0.5148	0.4621	0.5003	0.5542	0.6358	0.7245	0.7326	0.7678
	CTE 2013 [9], [10], [11]	ED	3280	3044	3370	3413	3662	3598	3774	3718	3522	3539	3337	3336
		TSS ED	2086	1922	1990	1696	1527	1343	1527	1673	1830	2114	2020	2127
		SC	0.6359	0.6313	0.5904	0.4968	0.4171	0.3731	0.4045	0.4499	0.5197	0.5975	0.6052	0.6375
Los Andes	NCL [2], [3], [4]	ED	3514	3332	3689	3787	4098	4089	4244	4128	3891	3806	3552	3066
		TSS ED	2705	2594	2573	2246	2016	1509	1826	1977	2172	2338	2441	2500
		SC	0.7697	0.7783	0.6975	0.5931	0.4918	0.3691	0.4302	0.4790	0.5581	0.6143	0.6872	0.8153
	CTE 2009 [5], [6], [7]	ED	3749	3555	3935	4040	4372	4361	4527	4403	4150	4060	3788	3271
		TSS ED	2908	2789	2768	2418	2170	1625	1966	2129	2338	2516	2625	2685
		SC	0.7757	0.7846	0.7033	0.5985	0.4964	0.3726	0.4343	0.4835	0.5633	0.6197	0.6930	0.8209
	CTE 2013 [9], [10], [11]	ED	4055	3845	4257	4370	4729	4718	4898	4763	4490	4392	4098	3539
		TSS ED	2666	2554	2516	2175	1936	1440	1746	1897	2096	2270	2385	2486
		SC	0.6573	0.6641	0.5910	0.4977	0.4094	0.3052	0.3565	0.3983	0.4668	0.5167	0.5820	0.7026
Ovalle	NCL [2], [3], [4]	ED	2833	2611	2891	2939	3135	3099	3222	3193	3033	3037	2864	2881
		TSS ED	1985	1802	1875	1594	1384	1227	1344	1510	1673	1958	1955	2042
		SC	0.7008	0.6902	0.6486	0.5424	0.4414	0.3958	0.4171	0.4730	0.5516	0.6446	0.6825	0.7087
	CTE 2009 [5], [6], [7]	ED	3022	2786	3084	3135	3344	3306	3437	3406	3236	3240	3055	3074
		TSS ED	2308	2097	2187	1870	1630	1448	1585	1778	1964	2286	2277	2374
		SC	0.7637	0.7530	0.7092	0.5965	0.4875	0.4379	0.4613	0.5221	0.6069	0.7057	0.7453	0.7723
	CTE 2013 [9], [10], [11]	ED	3269	3013	3336	3392	3617	3577	3718	3685	3500	3505	3305	3325
		TSS ED	2074	1881	1953	1651	1427	1264	1385	1559	1733	2037	2039	2134
		SC	0.6345	0.6243	0.5855	0.4869	0.3946	0.3533	0.3725	0.4231	0.4951	0.5812	0.6169	0.6418
Puente Alto	NCL [2], [3], [4]	ED	2677	2550	2823	2920	3212	3203	3310	3261	3062	2969	2779	2765
		TSS ED	2226	2028	2075	1674	1327	1069	1279	1522	1716	2032	2138	2227
		SC	0.8314	0.7955	0.7350	0.5732	0.4131	0.3337	0.3866	0.4669	0.5605	0.6843	0.7693	0.8055
	CTE 2009 [5], [6], [7]	ED	2856	2720	3011	3115	3427	3417	3530	3479	3266	3167	2964	2949
		TSS ED	2789	2564	2648	2191	1769	1434	1711	2022	2254	2619	2717	2808
		SC	0.9766	0.9427	0.8795	0.7032	0.5163	0.4197	0.4848	0.5813	0.6902	0.8270	0.9164	0.9521
	CTE 2013 [9], [10], [11]	ED	3089	2942	3258	3370	3707	3696	3819	3763	3533	3426	3207	3190
		TSS ED	2659	2427	2487	2015	1602	1292	1546	1837	2066	2439	2560	2663
		SC	0.8607	0.8247	0.7633	0.5978	0.4322	0.3495	0.4047	0.4881	0.5849	0.7118	0.7983	0.8348
San Bernardo	NCL [2], [3], [4]	ED	2677	2550	2823	2920	3212	3203	3310	3232	3062	3018	2779	2774
		TSS ED	2171	1967	2015	1601	1265	1008	1214	1432	1651	1965	2069	2162
		SC	0.8111	0.7713	0.7137	0.5483	0.3939	0.3147	0.3668	0.4430	0.5391	0.6511	0.7444	0.7793
	CTE 2009 [5], [6], [7]	ED	2856	2720	3011	3115	3427	3417	3530	3447	3266	3219	2964	2959
		TSS ED	2504	2276	2340	1878	1493	1192	1435	1688	1938	2293	2399	2500
		SC	0.8768	0.8370	0.7771	0.6028	0.4358	0.3488	0.4063	0.4897	0.5935	0.7124	0.8094	0.8448
	CTE 2013 [9], [10], [11]	ED	3089	2942	3258	3370	3707	3696	3819	3729	3533	3482	3207	3202
		TSS ED	2285	2063	2107	1660	1303	1036	1249	1477	1708	2045	2166	2270
		SC	0.7398	0.7011	0.6466	0.4924	0.3516	0.2803	0.3270	0.3959	0.4835	0.5873	0.6754	0.7089
San Felipe	NCL [2], [3], [4]	ED	2658	2532	2804	2911	3154	3194	3300	3203	3005	2950	2713	2833
		TSS ED	2096	1889	1938	1577	1282	1039	1242	1460	1641	1899	1975	2109
		SC	0.7885	0.7458	0.6913	0.5418	0.4065	0.3254	0.3764	0.4558	0.5461	0.6437	0.7278	0.7445
	CTE 2009 [5], [6], [7]	ED	2835	2701	2991	3105	3364	3407	3520	3416	3206	3146	2894	3022
		TSS ED	2711	2469	2557	2134	1765	1442	1718	2001	2223	2530	2590	2759
		SC	0.9565	0.9141	0.8551	0.6873	0.5245	0.4234	0.4880	0.5858	0.6934	0.8043	0.8948	0.9132
	CTE 2013 [9], [10], [11]	ED	3067	2922	3235	3359	3640	3685	3808	3696	3468	3404	3131	3269
		TSS ED	2544	2298	2362	1932	1576	1280	1529	1793	2011	2319	2404	2566
		SC	0.8295	0.7863	0.7301	0.5752	0.4331	0.3474	0.4014	0.4853	0.5799	0.6813	0.7677	0.7850
Santiago	NCL [2], [3], [4]	ED	2677	2550	2823	2920	3212	3203	3310	3242	3062	2979	2779	2726
		TSS ED	2210	1993	2043	1639	1288	1037	1241	1482	1681	1995	2099	2196
		SC	0.8255	0.7818	0.7235	0.5611	0.4011	0.3237	0.3748	0.4570	0.5491	0.6696	0.7553	0.8055
	CTE 2009 [5], [6], [7]	ED	2856	2720	3011	3115	3427	3417	3530	3458	3266	3177	2964	2907
		TSS ED	2772	2526	2612	2147	1719	1391	1661	1969	2211	2577	2673	2766
		SC	0.9707	0.9286	0.8673	0.6893	0.5017	0.4073	0.4704	0.5694	0.6770	0.8109	0.9017	0.9513
	CTE 2013 [9], [10], [11]	ED	3089	2942	3258	3370	3707	3696	3819	3741	3533	3437	3207	3145
		TSS ED	2640	2386	2448	1972	1556	1253	1499	1788	2025	2395	2515	2625
		SC	0.8547	0.8109	0.7516	0.5853	0.4197	0.3391	0.3924	0.4779	0.5731	0.6967	0.7841	0.8347
Talagante	NCL [2], [3], [4]	ED	2677	2550	2823	2920	3212	3203	3310	3242	3062	3018	2779	2774
		TSS ED	2156	1952	1996	1597	1263	990	1212	1422	1647	1941	2048	2144
		SC	0.8052	0.7657	0.7069	0.5467	0.3932	0.3092	0.3663	0.4386	0.5379	0.6431	0.7368	0.7727
	CTE 2009 [5], [6], [7]	ED	2856	2720	3011	3115	3427	3417	3530	3458	3266	3219	2964	2959
		TSS ED	2487	2261	2319	1872	1491	1171	1432	1676	1934	2266	2376	2480
		SC	0.8708	0.8312	0.7701	0.6011	0.4351	0.3427	0.4057	0.4848	0.5923	0.7039	0.8015	0.8381
	CTE 2013 [9], [10], [11]	ED	3089	2942	3258	3370	3707	3696	3819	3741	3533	3482	3207	3202
		TSS ED	2268	2047	2086	1654	1301	1018	1247	1466	1705	2019	2143	2250
		SC	0.7341	0.6958	0.6403	0.4909	0.3510	0.2754	0.3265	0.3919	0.4825	0.5799	0.6682	0.7027

**Table 4:** Domestic hot water energy demand (ED), in kWh; domestic hot water energy demand met by the thermal solar system (TSS ED), in kWh; and solar contribution (SC) according to commune, regulation and month in solar climate zone C.

Commune	Regulation	Parameter	Jan	Feb	Mar	Apr	May	Jun	Jul	Aug	Sep	Oct	Nov	Dec
Angol	NCL [2], [3], [4]	ED	3154	2963	3281	3297	3505	3476	3631	3553	3391	3417	3222	3232
		TSS ED	2192	2023	2061	1605	1159	949	1007	1344	1641	1953	1956	2071
		SC	0.6950	0.6828	0.6281	0.4867	0.3307	0.2730	0.2773	0.3783	0.4838	0.5716	0.6070	0.6408
	CTE 2009 [5], [6], [7]	ED	3364	3161	3499	3517	3738	3708	3873	3790	3618	3645	3437	3447
		TSS ED	1895	1744	1767	1359	972	794	842	1129	1388	1665	1673	1779
		SC	0.5631	0.5518	0.5050	0.3864	0.2601	0.2141	0.2174	0.2980	0.3837	0.4568	0.4869	0.5161
	CTE 2013 [9], [10], [11]	ED	3640	3419	3786	3805	4044	4011	4190	4100	3914	3943	3718	3729
		TSS ED	2116	1949	1976	1523	1092	893	947	1268	1556	1863	1872	1989
		SC	0.5815	0.5700	0.5221	0.4003	0.2700	0.2225	0.2259	0.3092	0.3975	0.4726	0.5035	0.5333
Cauquenes	NCL [2], [3], [4]	ED	2989	2787	3086	3109	3310	3260	3407	3358	3203	3222	3024	3037
		TSS ED	2278	2022	2052	1609	1092	851	985	1326	1668	2011	2100	2173
		SC	0.7622	0.7254	0.6650	0.5175	0.3301	0.2611	0.2892	0.3949	0.5208	0.6240	0.6945	0.7154
	CTE 2009 [5], [6], [7]	ED	3188	2973	3292	3316	3530	3477	3634	3582	3417	3437	3226	3240
		TSS ED	2324	2060	2087	1630	1102	857	993	1340	1690	2042	2137	2213
		SC	0.7290	0.6929	0.6340	0.4915	0.3121	0.2465	0.2732	0.3739	0.4946	0.5942	0.6627	0.6832
	CTE 2013 [9], [10], [11]	ED	3449	3216	3561	3587	3819	3761	3932	3876	3696	3718	3490	3505
		TSS ED	2218	1960	1979	1533	1032	803	929	1255	1589	1930	2029	2105
		SC	0.6431	0.6093	0.5557	0.4275	0.2702	0.2136	0.2364	0.3238	0.4298	0.5191	0.5815	0.6007
Chillán	NCL [2], [3], [4]	ED	2998	2761	3057	3099	3261	3231	3368	3310	3194	3203	3005	3057
		TSS ED	2118	1836	1872	1486	984	790	875	1201	1530	1859	1917	2011
		SC	0.7066	0.6650	0.6123	0.4794	0.3016	0.2444	0.2598	0.3629	0.4792	0.5804	0.6378	0.6578
	CTE 2009 [5], [6], [7]	ED	3198	2945	3261	3306	3479	3447	3593	3530	3407	3416	3206	3261
		TSS ED	2317	2010	2050	1630	1079	865	959	1318	1679	2038	2099	2201
		SC	0.7246	0.6825	0.6287	0.4930	0.3101	0.2511	0.2670	0.3733	0.4929	0.5965	0.6549	0.6752
	CTE 2013 [9], [10], [11]	ED	3460	3186	3527	3577	3763	3729	3887	3819	3685	3696	3468	3527
		TSS ED	2211	1912	1944	1534	1011	811	899	1235	1579	1926	1993	2093
		SC	0.6391	0.6000	0.5511	0.4288	0.2687	0.2176	0.2312	0.3234	0.4284	0.5213	0.5746	0.5933
Concepción	NCL [2], [3], [4]	ED	2998	2796	3096	3090	3290	3231	3388	3339	3184	3212	3043	3028
		TSS ED	2232	1908	1972	1573	1022	845	905	1288	1618	1986	2034	2119
		SC	0.7446	0.6825	0.6371	0.5092	0.3106	0.2614	0.2671	0.3856	0.5080	0.6182	0.6684	0.7000
	CTE 2009 [5], [6], [7]	ED	3198	2982	3302	3296	3510	3447	3614	3562	3396	3427	3246	3229
		TSS ED	1944	1649	1697	1338	859	709	759	1085	1375	1704	1754	1836
		SC	0.6079	0.5530	0.5140	0.4061	0.2448	0.2057	0.2101	0.3047	0.4047	0.4972	0.5405	0.5686
	CTE 2013 [9], [10], [11]	ED	3460	3227	3572	3566	3797	3729	3909	3853	3674	3707	3511	3494
		TSS ED	2170	1843	1898	1499	965	798	854	1218	1540	1906	1961	2051
		SC	0.6271	0.5711	0.5312	0.4205	0.2543	0.2139	0.2184	0.3161	0.4191	0.5141	0.5584	0.5871
Coquimbo	NCL [2], [3], [4]	ED	2852	2620	2901	2902	3086	3033	3144	3135	2996	3047	2883	2901
		TSS ED	1695	1591	1669	1434	1251	1112	1239	1383	1508	1746	1681	1774
		SC	0.5944	0.6071	0.5755	0.4943	0.4053	0.3667	0.3941	0.4412	0.5035	0.5729	0.5830	0.6114
	CTE 2009 [5], [6], [7]	ED	3042	2795	3094	3095	3292	3236	3354	3344	3196	3250	3075	3094
		TSS ED	2150	2016	2122	1838	1614	1440	1602	1782	1934	2224	2137	2247
		SC	0.7067	0.7213	0.6859	0.5939	0.4904	0.4450	0.4775	0.5331	0.6053	0.6842	0.6950	0.7261
	CTE 2013 [9], [10], [11]	ED	3291	3024	3348	3348	3561	3500	3628	3617	3457	3516	3327	3348
		TSS ED	2073	1945	2043	1759	1537	1369	1524	1699	1850	2137	2056	2168
		SC	0.6299	0.6431	0.6102	0.5255	0.4317	0.3910	0.4200	0.4698	0.5353	0.6078	0.6182	0.6476
Curicó	NCL [2], [3], [4]	ED	2843	2699	2989	3109	3388	3410	3524	3446	3241	3174	2930	3018
		TSS ED	2140	2009	1987	1600	1222	811	1038	1243	1504	1706	1840	2034
		SC	0.7530	0.7442	0.6649	0.5147	0.3607	0.2379	0.2945	0.3608	0.4642	0.5375	0.6279	0.6739
	CTE 2009 [5], [6], [7]	ED	3032	2879	3188	3316	3614	3638	3759	3676	3457	3385	3125	3219
		TSS ED	2339	2196	2175	1755	1341	890	1139	1365	1651	1870	2015	2226
		SC	0.7714	0.7628	0.6822	0.5293	0.3712	0.2446	0.3030	0.3713	0.4776	0.5525	0.6446	0.6915
	CTE 2013 [9], [10], [11]	ED	3280	3115	3449	3587	3909	3935	4066	3977	3740	3662	3381	3482
		TSS ED	2242	2101	2070	1654	1256	833	1065	1277	1551	1765	1912	2119
		SC	0.6834	0.6745	0.6001	0.4610	0.3213	0.2116	0.2618	0.3212	0.4147	0.4820	0.5656	0.6084
La Ligua	NCL [2], [3], [4]	ED	2473	2365	2619	2741	2989	2996	3125	3037	2836	2765	2572	2609
		TSS ED	1839	1616	1649	1307	1068	868	996	1250	1434	1657	1748	1834
		SC	0.7439	0.6831	0.6298	0.4769	0.3574	0.2898	0.3188	0.4117	0.5057	0.5995	0.6798	0.7030
	CTE 2009 [5], [6], [7]	ED	2637	2523	2793	2924	3188	3196	3333	3240	3025	2949	2743	2783
		TSS ED	2007	1766	1803	1432	1170	951	1092	1371	1571	1813	1911	2004
		SC	0.7611	0.6998	0.6456	0.4897	0.3671	0.2975	0.3275	0.4231	0.5194	0.6149	0.6965	0.7200
	CTE 2013 [9], [10], [11]	ED	2853	2729	3022	3163	3449	3457	3606	3505	3272	3190	2968	3011
		TSS ED	1932	1690	1720	1353	1101	894	1026	1290	1485	1724	1827	1920
		SC	0.6770	0.6191	0.5693	0.4278	0.3194	0.2586	0.2845	0.3682	0.4538	0.5405	0.6157	0.6378
Linares	NCL [2], [3], [4]	ED	2813	2673	2959	3062	3300	3307	3427	3349	3165	3125	2892	2891
		TSS ED	2094	1916	1916	1478	1040	742	904	1202	1489	1791	1880	1982
		SC	0.7443	0.7167	0.6474	0.4827	0.3151	0.2244	0.2637	0.3589	0.4706	0.5732	0.6499	0.6857
	CTE 2009 [5], [6], [7]	ED	3001	2851	3157	3266	3520	3527	3655	3572	3376	3333	3085	3084
		TSS ED	2288	2095	2097	1621	1141	813	991	1319	1634	1963	2057	2168
		SC	0.7625	0.7348	0.6644	0.4963	0.3240	0.2305	0.2711	0.3693	0.4840	0.5889	0.6669	0.7031
	CTE 2013 [9], [10], [11]	ED	3246	3084	3415	3533	3808	3816	3954	3864	3653	3606	3337	3336
		TSS ED	2193	2001	1994	1526	1069	762	928	1236	1537	1856	1956	2068
		SC	0.6754	0.6488	0.5840	0.4320	0.2806	0.1998	0.2346	0.3198	0.4207	0.5148	0.5862	0.6199
Los Ángeles	NCL [2], [3], [4]	ED	3028	2796	3096	3118	3310	3269	3417	3358	3212	3232	3043	3076
		TSS ED	2243	1945	1981	1554	1040	841	918	1265	1616	1983	2031	2130
		SC	0.7409	0.6955	0.6398	0.4984	0.3142	0.2574	0.2686	0.3768	0.5031	0.6136	0.6674	0.6923
	CTE 2009 [5], [6], [7]	ED	3229	2982	3302	3326	3530	3487	3645	3582	3427	3447	3246	3281
		TSS ED	1952	1683	1705	1321	874	706	770	1066	1372	1700	1752	1842
		SC	0.6043	0.5643	0.5163	0.3970	0.2476	0.2025	0.2112	0.2975	0.4005	0.4932	0.5397	0.5615
	CTE 2013 [9], [10], [11]	ED	3494	3227	3572	3598	3819	3772	3943	3876	3707	3729	3511	3550
		TSS ED	2178	1880	1906	1479	982	794	866	1196	1537	1902	1958	2058
		SC	0.6235	0.5827	0.5335	0.4111	0.2571	0.2105	0.2195	0.3087	0.4147	0.5100	0.5575	0.5798
Melipilla	NCL [2], [3], [4]	ED	2482	2374	2628	2732	3008	2996	3115	3018	2873	2823	2591	2531
		TSS ED	1755	1563	1605	1264	974	782	938	1172	1362	1586	1661	1716
		SC	0.7068	0.6582	0.6108	0.4627	0.3239	0.2611	0.3010	0.3883	0.4739	0.5617	0.6410	0.6781
	CTE 2009 [5], [6], [7]	ED	2648	2532	2804	2914	3209	3196	3323	3219	3065	3011	2763	2700
		TSS ED	1934	1724	1773	1399	1079	866	1039	1298	1508	1754	1833	1893
		SC	0.7302	0.6809	0.6324	0.4801	0.3364	0.2710	0.3127	0.4033	0.4920	0.5823	0.6634	0.7011
	CTE 2013 [9], [10], [11]	ED	2865	2739	3033	3153	3471	3457	3595	3482	3316	3258	2990	2921
		TSS ED	1991	1772	1820	1431	1103	885	1061	1326	1542	1797	1883	1947
		SC	0.6951	0.6469	0.6001	0.4540	0.3176	0.2560	0.2951	0.3808	0.4650	0.5515	0.6298	0.6667
Pichilemu	NCL [2], [3], [4]	ED	2560	2436	2697	2770	3037	3033	3154	3086	2892	2843	2628	2609
		TSS ED	1919	1728	1772	1373	988	722	923	1188	1439	1691	1789	1855
		SC	0.7495	0.7093	0.6570	0.4959	0.3254	0.2381	0.2927	0.3850	0.4976	0.5951	0.6808	0.7112
	CTE 2009 [5], [6], [7]	ED	2731	2598	2876	2954	3240	3236	3364	3292	3085	3032	2804	2783
		TSS ED	2095	1888	1937	1504	1083	790	1012	1302	1577	1851	1956	2027
		SC	0.7670	0.7266	0.6735	0.5092	0.3342	0.2442	0.3007	0.3957	0.5112	0.6106	0.6977	0.7283
	CTE 2013 [9], [10], [11]	ED	2954	2811	3112	3196	3505	3500	3640	3561	3337	3280	3033	3011
		TSS ED	2014	1808	1849	1422	1018	744	950	1225	1489	1759	1869	1943
		SC	0.6818	0.6433	0.5943	0.4450	0.2905	0.2125	0.2611	0.3439	0.4463	0.5362	0.6164	0.6454
Quillota	NCL [2], [3], [4]	ED	2463	2339	2589	2694	2979	2977	3096	2998	2836	2784	2553	2628
		TSS ED	1784	1549	1620	1292	1030	838	990	1266	1450	1617	1683	1768
		SC	0.7245	0.6625	0.6257	0.4797	0.3456	0.2815	0.3197	0.4223	0.5113	0.5810	0.6590	0.6728
	CTE 2009 [5], [6], [7]	ED	2627	2495	2762	2874	3177	3175	3302	3198	3025	2970	2723	2804
		TSS ED	1679	1450	1513	1195	946	768	908	1166	1342	1504	1574	1656
		SC	0.6390	0.5812	0.5477	0.4159	0.2976	0.2418	0.2749	0.3647	0.4436	0.5064	0.5779	0.5905
	CTE 2013 [9], [10], [11]	ED	2842	2699	2988	3109	3437	3435	3572	3460	3272	3213	2946	3033
		TSS ED	1872	1619	1690	1339	1062	863	1019	1307	1502	1682	1757	1848
		SC	0.6587	0.5998	0.5656	0.4305	0.3088	0.2512	0.2854	0.3779	0.4590	0.5234	0.5964	0.6093
Quilpué	NCL [2], [3], [4]	ED	2619	2480	2745	2845	3135	3128	3251	3183	2986	2940	2704	2551
		TSS ED	1814	1606	1649	1310	1024	824	997	1202	1370	1613	1700	1764
		SC	0.6928	0.6477	0.6008	0.4604	0.3266	0.2634	0.3066	0.3776	0.4586	0.5487	0.6288	0.6916
	CTE 2009 [5], [6], [7]	ED	2793	2645	2928	3035	3344	3336	3468	3395	3185	3136	2884	2721
		TSS ED	2001	1774	1823	1450	1135	913	1105	1333	1518	1785	1878	1945
		SC	0.7164	0.6706	0.6224	0.4780	0.3394	0.2736	0.3187	0.3925	0.4764	0.5692	0.6512	0.7150
	CTE 2013 [9], [10], [11]	ED	3022	2861	3168	3283	3617	3609	3752	3673	3446	3392	3120	2943
		TSS ED	2058	1821	1869	1483	1158	932	1127	1360	1550	1827	1927	2001
		SC	0.6811	0.6364	0.5901	0.4516	0.3202	0.2582	0.3005	0.3702	0.4499	0.5386	0.6177	0.6800
Rancagua	NCL [2], [3], [4]	ED	2677	2550	2823	2920	3212	3203	3310	3261	3062	3018	2779	2726
		TSS ED	1987	1792	1826	1448	1108	841	1047	1280	1500	1774	1882	1950
		SC	0.7424	0.7029	0.6467	0.4959	0.3449	0.2627	0.3163	0.3924	0.4899	0.5879	0.6773	0.7152
	CTE 2009 [5], [6], [7]	ED	2856	2720	3011	3115	3427	3417	3530	3479	3266	3219	2964	2907
		TSS ED	2171	1959	1997	1587	1215	922	1148	1404	1645	1943	2059	2131
		SC	0.7601	0.7204	0.6633	0.5096	0.3546	0.2699	0.3253	0.4036	0.5037	0.6037	0.6946	0.7328
	CTE 2013 [9], [10], [11]	ED	3089	2942	3258	3370	3707	3696	3819	3763	3533	3482	3207	3145
		TSS ED	2083	1873	1902	1498	1140	865	1076	1318	1550	1841	1964	2040
		SC	0.6743	0.6365	0.5839	0.4445	0.3075	0.2340	0.2818	0.3501	0.4387	0.5288	0.6124	0.6485
San Antonio	NCL [2], [3], [4]	ED	2473	2365	2619	2723	2979	2958	3105	3008	2836	2813	2581	2521
		TSS ED	1759	1554	1602	1261	977	797	953	1198	1392	1603	1672	1722
		SC	0.7116	0.6570	0.6119	0.4632	0.3281	0.2693	0.3069	0.3983	0.4908	0.5696	0.6479	0.6830
	CTE 2009 [5], [6], [7]	ED	2637	2523	2793	2904	3177	3155	3312	3209	3025	3001	2753	2689
		TSS ED	1654	1453	1494	1165	897	730	874	1102	1286	1489	1563	1615
		SC	0.6270	0.5761	0.5350	0.4012	0.2824	0.2313	0.2638	0.3435	0.4253	0.4961	0.5676	0.6004
	CTE 2013 [9], [10], [11]	ED	2853	2729	3022	3142	3437	3413	3583	3471	3272	3246	2979	2909
		TSS ED	1845	1623	1670	1305	1008	821	981	1236	1440	1665	1745	1802
		SC	0.6465	0.5946	0.5526	0.4154	0.2931	0.2404	0.2739	0.3561	0.4402	0.5129	0.5859	0.6194
San Fernando	NCL [2], [3], [4]	ED	2911	2787	3086	3212	3485	3505	3641	3563	3326	3251	3015	2969
		TSS ED	2212	2099	2038	1701	1386	928	1136	1291	1517	1679	1859	2057
		SC	0.7600	0.7531	0.6603	0.5294	0.3978	0.2647	0.3120	0.3624	0.4561	0.5165	0.6167	0.6928
	CTE 2009 [5], [6], [7]	ED	3105	2973	3292	3427	3717	3738	3884	3800	3547	3468	3216	3167
		TSS ED	2418	2295	2231	1866	1522	1018	1248	1418	1665	1842	2037	2250
		SC	0.7787	0.7720	0.6778	0.5445	0.4096	0.2724	0.3212	0.3732	0.4694	0.5312	0.6334	0.7106
	CTE 2013 [9], [10], [11]	ED	3359	3216	3561	3707	4022	4044	4201	4111	3837	3752	3479	3426
		TSS ED	2316	2195	2120	1757	1425	951	1164	1326	1562	1735	1930	2145
		SC	0.6897	0.6824	0.5954	0.4741	0.3544	0.2351	0.2772	0.3224	0.4071	0.4625	0.5548	0.6261
Talca	NCL [2], [3], [4]	ED	2804	2655	2940	2986	3183	3137	3281	3232	3081	3086	2854	2804
		TSS ED	2142	1949	1980	1507	1000	672	932	1243	1557	1835	1962	2040
		SC	0.7641	0.7339	0.6735	0.5048	0.3141	0.2144	0.2841	0.3845	0.5054	0.5946	0.6875	0.7278
	CTE 2009 [5], [6], [7]	ED	2991	2832	3136	3185	3395	3346	3499	3447	3286	3292	3045	2991
		TSS ED	1875	1697	1714	1284	842	566	784	1049	1325	1574	1700	1779
		SC	0.6268	0.5991	0.5465	0.4031	0.2480	0.1691	0.2239	0.3043	0.4031	0.4781	0.5585	0.5947
	CTE 2013 [9], [10], [11]	ED	3235	3064	3392	3446	3673	3620	3786	3729	3555	3561	3294	3235
		TSS ED	2091	1894	1915	1438	946	637	881	1177	1484	1761	1900	1985
		SC	0.6464	0.6182	0.5644	0.4174	0.2576	0.1760	0.2327	0.3157	0.4174	0.4944	0.5767	0.6137

**Table 5:** Domestic hot water energy demand (ED), in kWh; domestic hot water energy demand met by the thermal solar system (TSS ED), in kWh; and solar contribution (SC) according to commune, regulation and month in solar climate zone D.

Commune	Regulation	Parameter	Jan	Feb	Mar	Apr	May	Jun	Jul	Aug	Sep	Oct	Nov	Dec
Chile Chico	NCL [2], [3], [4]	ED	3427	3227	3573	3618	3894	3919	4050	3972	3749	3719	3505	3524
		TSS ED	2048	1846	1850	1345	923	741	866	1230	1605	1862	1867	1918
		SC	0.5978	0.5720	0.5177	0.3718	0.2369	0.1892	0.2138	0.3096	0.4281	0.5008	0.5326	0.5442
	CTE 2009 [5], [6], [7]	ED	3655	3442	3811	3859	4154	4180	4320	4237	3999	3967	3738	3759
		TSS ED	1896	1705	1703	1229	839	673	786	1120	1470	1712	1720	1769
		SC	0.5188	0.4953	0.4469	0.3186	0.2020	0.1610	0.1820	0.2643	0.3674	0.4317	0.4601	0.4706
	CTE 2013 [9], [10], [11]	ED	3954	3724	4123	4174	4493	4522	4673	4583	4327	4291	4044	4066
		TSS ED	2120	1907	1907	1379	943	758	884	1257	1647	1917	1925	1980
		SC	0.5362	0.5121	0.4625	0.3304	0.2099	0.1675	0.1892	0.2744	0.3807	0.4468	0.4760	0.4868
Coyhaique	NCL [2], [3], [4]	ED	3339	3139	3475	3523	3787	3815	3943	3874	3655	3631	3420	3407
		TSS ED	2048	1855	1838	1369	938	769	906	1276	1664	1927	1944	2007
		SC	0.6133	0.5911	0.5289	0.3886	0.2477	0.2015	0.2299	0.3294	0.4551	0.5306	0.5683	0.5892
	CTE 2009 [5], [6], [7]	ED	3562	3348	3707	3758	4039	4070	4205	4133	3899	3873	3648	3634
		TSS ED	1899	1717	1695	1253	854	699	824	1164	1526	1775	1795	1858
		SC	0.5331	0.5127	0.4572	0.3334	0.2114	0.1717	0.1959	0.2816	0.3913	0.4583	0.4922	0.5112
	CTE 2013 [9], [10], [11]	ED	3853	3622	4010	4066	4370	4403	4550	4471	4218	4190	3946	3932
		TSS ED	2122	1920	1897	1405	960	786	927	1306	1710	1987	2008	2077
		SC	0.5508	0.5299	0.4730	0.3456	0.2197	0.1786	0.2037	0.2922	0.4053	0.4742	0.5089	0.5284
La Unión	NCL [2], [3], [4]	ED	3125	2910	3222	3241	3436	3420	3543	3495	3354	3368	3175	3203
		TSS ED	1891	1804	1650	1290	938	708	823	1133	1352	1537	1591	1790
		SC	0.6050	0.6198	0.5120	0.3981	0.2729	0.2071	0.2323	0.3242	0.4031	0.4564	0.5012	0.5589
	CTE 2009 [5], [6], [7]	ED	3333	3104	3437	3457	3665	3648	3780	3728	3577	3593	3386	3416
		TSS ED	1755	1675	1523	1184	856	646	750	1036	1240	1414	1467	1656
		SC	0.5265	0.5395	0.4430	0.3424	0.2336	0.1770	0.1986	0.2779	0.3465	0.3934	0.4332	0.4848
	CTE 2013 [9], [10], [11]	ED	3606	3358	3718	3740	3965	3946	4089	4033	3870	3887	3664	3696
		TSS ED	1962	1872	1704	1327	962	727	844	1163	1390	1584	1643	1853
		SC	0.5440	0.5574	0.4584	0.3549	0.2427	0.1841	0.2064	0.2884	0.3592	0.4075	0.4484	0.5013
Lebu	NCL [2], [3], [4]	ED	3008	2805	3105	3109	3300	3250	3397	3349	3203	3212	3052	3144
		TSS ED	1748	1543	1618	1283	872	705	746	1038	1291	1540	1560	1660
		SC	0.5810	0.5501	0.5210	0.4127	0.2641	0.2168	0.2196	0.3101	0.4030	0.4794	0.5110	0.5279
	CTE 2009 [5], [6], [7]	ED	3209	2992	3312	3316	3520	3467	3624	3572	3417	3427	3256	3354
		TSS ED	1772	1563	1638	1296	877	709	750	1046	1303	1557	1578	1680
		SC	0.5522	0.5224	0.4944	0.3907	0.2492	0.2044	0.2070	0.2929	0.3814	0.4544	0.4848	0.5010
	CTE 2013 [9], [10], [11]	ED	3471	3237	3583	3587	3808	3750	3920	3864	3696	3707	3522	3628
		TSS ED	1980	1747	1832	1452	986	797	844	1174	1460	1743	1766	1879
		SC	0.5703	0.5399	0.5111	0.4047	0.2588	0.2125	0.2152	0.3039	0.3951	0.4702	0.5013	0.5179
Osorno	NCL [2], [3], [4]	ED	3144	2919	3232	3231	3388	3410	3524	3475	3326	3378	3175	3193
		TSS ED	1860	1791	1604	1279	915	669	791	1116	1339	1524	1561	1764
		SC	0.5914	0.6137	0.4962	0.3959	0.2701	0.1961	0.2244	0.3211	0.4025	0.4511	0.4918	0.5524
	CTE 2009 [5], [6], [7]	ED	3354	3114	3447	3447	3614	3638	3759	3707	3547	3603	3386	3406
		TSS ED	1724	1663	1479	1173	836	610	721	1020	1227	1401	1439	1632
		SC	0.5142	0.5340	0.4290	0.3405	0.2312	0.1677	0.1918	0.2751	0.3460	0.3888	0.4249	0.4790
	CTE 2013 [9], [10], [11]	ED	3628	3369	3729	3729	3909	3935	4066	4010	3837	3898	3664	3685
		TSS ED	1928	1858	1656	1316	939	687	811	1145	1376	1570	1611	1825
		SC	0.5314	0.5517	0.4440	0.3529	0.2403	0.1745	0.1995	0.2856	0.3587	0.4027	0.4398	0.4954
Puerto Montt	NCL [2], [3], [4]	ED	3193	2954	3271	3307	3505	3486	3621	3592	3420	3436	3231	3232
		TSS ED	1931	1857	1671	1324	911	695	804	1147	1365	1605	1658	1842
		SC	0.6047	0.6284	0.5109	0.4003	0.2600	0.1995	0.2220	0.3192	0.3992	0.4670	0.5131	0.5700
	CTE 2009 [5], [6], [7]	ED	3406	3151	3489	3527	3738	3718	3863	3832	3648	3665	3447	3447
		TSS ED	1654	1593	1420	1115	763	582	672	961	1149	1357	1408	1573
		SC	0.4857	0.5054	0.4069	0.3161	0.2041	0.1565	0.1740	0.2507	0.3149	0.3702	0.4084	0.4562
	CTE 2013 [9], [10], [11]	ED	3685	3409	3774	3816	4044	4022	4179	4145	3946	3965	3729	3729
		TSS ED	1850	1781	1590	1251	858	655	756	1079	1289	1521	1577	1760
		SC	0.5022	0.5224	0.4213	0.3278	0.2121	0.1629	0.1810	0.2603	0.3265	0.3835	0.4229	0.4720
Temuco	NCL [2], [3], [4]	ED	3047	2840	3144	3175	3358	3326	3475	3427	3278	3290	3109	3125
		TSS ED	1773	1690	1686	1281	916	723	796	1094	1293	1512	1532	1669
		SC	0.5818	0.5949	0.5362	0.4036	0.2728	0.2175	0.2290	0.3193	0.3944	0.4595	0.4929	0.5341
	CTE 2009 [5], [6], [7]	ED	3250	3029	3354	3386	3582	3547	3707	3655	3497	3510	3316	3333
		TSS ED	1797	1713	1707	1293	922	727	800	1103	1305	1528	1550	1690
		SC	0.5529	0.5655	0.5090	0.3820	0.2574	0.2050	0.2159	0.3016	0.3731	0.4354	0.4674	0.5070
	CTE 2013 [9], [10], [11]	ED	3516	3277	3628	3664	3876	3837	4010	3954	3783	3797	3587	3606
		TSS ED	2008	1914	1909	1450	1036	818	900	1237	1463	1711	1734	1890
		SC	0.5710	0.5839	0.5261	0.3957	0.2673	0.2132	0.2244	0.3129	0.3866	0.4506	0.4834	0.5241
Valdivia	NCL [2], [3], [4]	ED	3047	2840	3144	3184	3349	3335	3446	3427	3278	3290	3099	3105
		TSS ED	1913	1830	1693	1313	943	722	821	1131	1364	1556	1612	1821
		SC	0.6279	0.6444	0.5384	0.4125	0.2816	0.2166	0.2382	0.3299	0.4161	0.4729	0.5202	0.5863
	CTE 2009 [5], [6], [7]	ED	3250	3029	3354	3396	3572	3557	3676	3655	3497	3510	3306	3312
		TSS ED	1780	1703	1566	1206	862	659	749	1034	1252	1433	1489	1688
		SC	0.5475	0.5621	0.4668	0.3551	0.2413	0.1853	0.2038	0.2829	0.3581	0.4082	0.4503	0.5097
	CTE 2013 [9], [10], [11]	ED	3516	3277	3628	3674	3864	3848	3977	3954	3783	3797	3577	3583
		TSS ED	1988	1902	1752	1352	969	742	843	1161	1404	1605	1666	1888
		SC	0.5655	0.5805	0.4828	0.3680	0.2506	0.1928	0.2119	0.2936	0.3711	0.4227	0.4659	0.5268
Valparaiso	NCL [2], [3], [4]	ED	2521	2365	2619	2723	3008	3005	3154	3057	2864	2813	2581	2482
		TSS ED	1601	1398	1436	1135	870	717	846	1073	1225	1432	1489	1555
		SC	0.6349	0.5910	0.5483	0.4168	0.2892	0.2387	0.2683	0.3510	0.4277	0.5089	0.5769	0.6262
	CTE 2009 [5], [6], [7]	ED	2689	2523	2793	2904	3209	3206	3364	3261	3055	3001	2753	2648
		TSS ED	1627	1419	1456	1147	876	722	852	1082	1238	1450	1511	1580
		SC	0.6049	0.5624	0.5211	0.3948	0.2732	0.2252	0.2533	0.3320	0.4052	0.4831	0.5487	0.5965
	CTE 2013 [9], [10], [11]	ED	2909	2729	3022	3142	3471	3468	3640	3527	3305	3246	2979	2865
		TSS ED	1815	1585	1627	1285	984	812	957	1214	1387	1622	1688	1763
		SC	0.6240	0.5806	0.5384	0.4089	0.2836	0.2341	0.2630	0.3442	0.4196	0.4995	0.5666	0.6154

**Table 6:** Domestic hot water energy demand (ED), in kWh; domestic hot water energy demand met by the thermal solar system (TSS ED), in kWh; and solar contribution (SC) according to commune, regulation and month in solar climate zone E.

Commune	Regulation	Parameter	Jan	Feb	Mar	Apr	May	Jun	Jul	Aug	Sep	Oct	Nov	Dec
Aysén	NCL [2], [3], [4]	ED	3105	2928	3242	3288	3553	3580	3699	3631	3429	3397	3184	3183
		TSS ED	1515	1410	1401	1015	676	541	633	943	1247	1477	1415	1495
		SC	0.4878	0.4816	0.4321	0.3089	0.1903	0.1512	0.1712	0.2596	0.3636	0.4348	0.4445	0.4697
	CTE 2009 [5], [6], [7]	ED	3312	3123	3458	3507	3790	3819	3946	3873	3658	3624	3396	3395
		TSS ED	1532	1426	1415	1023	679	543	636	948	1257	1492	1430	1512
		SC	0.4626	0.4565	0.4091	0.2917	0.1793	0.1423	0.1612	0.2448	0.3437	0.4117	0.4210	0.4452
	CTE 2013 [9], [10], [11]	ED	3583	3379	3741	3794	4100	4131	4269	4190	3957	3920	3674	3673
		TSS ED	1715	1596	1585	1148	765	612	716	1065	1410	1671	1601	1692
		SC	0.4785	0.4723	0.4236	0.3026	0.1865	0.1482	0.1677	0.2543	0.3562	0.4263	0.4358	0.4606
Castro	NCL [2], [3], [4]	ED	3193	2972	3290	3278	3436	3448	3582	3534	3373	3388	3231	3242
		TSS ED	1572	1539	1342	1035	733	520	618	898	1131	1322	1384	1492
		SC	0.4923	0.5179	0.4079	0.3157	0.2133	0.1508	0.1724	0.2542	0.3352	0.3902	0.4284	0.4603
	CTE 2009 [5], [6], [7]	ED	3406	3170	3510	3497	3665	3678	3821	3769	3597	3614	3447	3458
		TSS ED	1590	1557	1355	1043	737	522	621	904	1139	1334	1398	1508
		SC	0.4668	0.4913	0.3860	0.2982	0.2010	0.1419	0.1624	0.2398	0.3167	0.3691	0.4056	0.4361
	CTE 2013 [9], [10], [11]	ED	3685	3429	3797	3783	3965	3979	4134	4078	3892	3909	3729	3741
		TSS ED	1779	1742	1518	1170	829	588	699	1016	1278	1495	1566	1688
		SC	0.4828	0.5080	0.3999	0.3093	0.2090	0.1478	0.1690	0.2490	0.3285	0.3825	0.4200	0.4513
Chaitén	NCL [2], [3], [4]	ED	3232	3025	3349	3382	3621	3655	3777	3709	3514	3495	3297	3300
		TSS ED	1569	1543	1445	1079	770	613	631	951	1222	1427	1466	1470
		SC	0.4854	0.5101	0.4314	0.3192	0.2126	0.1678	0.1670	0.2564	0.3479	0.4084	0.4447	0.4454
	CTE 2009 [5], [6], [7]	ED	3447	3226	3572	3608	3863	3899	4029	3956	3748	3728	3517	3520
		TSS ED	1586	1561	1459	1087	774	616	634	957	1232	1440	1481	1485
		SC	0.4601	0.4838	0.4084	0.3014	0.2004	0.1580	0.1573	0.2418	0.3287	0.3864	0.4211	0.4218
	CTE 2013 [9], [10], [11]	ED	3729	3490	3864	3903	4179	4218	4359	4280	4055	4033	3805	3808
		TSS ED	1775	1746	1634	1220	870	693	713	1075	1382	1614	1659	1663
		SC	0.4760	0.5003	0.4229	0.3127	0.2083	0.1644	0.1637	0.2511	0.3408	0.4002	0.4359	0.4366
Cochrane	NCL [2], [3], [4]	ED	3427	3218	3563	3599	3884	3900	4030	3943	3740	3709	3476	3495
		TSS ED	1701	1563	1599	1136	796	606	735	1039	1394	1603	1563	1590
		SC	0.4963	0.4858	0.4488	0.3156	0.2049	0.1553	0.1823	0.2635	0.3726	0.4323	0.4495	0.4550
	CTE 2009 [5], [6], [7]	ED	3655	3433	3800	3839	4143	4160	4299	4205	3989	3956	3708	3728
		TSS ED	1720	1580	1615	1144	800	608	738	1045	1405	1619	1578	1606
		SC	0.4705	0.4603	0.4250	0.2979	0.1930	0.1462	0.1716	0.2485	0.3522	0.4091	0.4256	0.4309
	CTE 2013 [9], [10], [11]	ED	3954	3714	4111	4153	4482	4501	4651	4550	4316	4280	4011	4033
		TSS ED	1924	1768	1809	1283	899	685	830	1174	1575	1813	1767	1799
		SC	0.4866	0.4762	0.4399	0.3091	0.2007	0.1521	0.1785	0.2580	0.3650	0.4236	0.4406	0.4460
Porvenir	NCL [2], [3], [4]	ED	3602	3341	3699	3684	3952	3928	4050	4001	3787	3806	3589	3709
		TSS ED	1917	1638	1677	1187	750	384	519	855	1498	1741	1969	1956
		SC	0.5323	0.4901	0.4533	0.3222	0.1899	0.0977	0.1281	0.2138	0.3955	0.4574	0.5487	0.5273
	CTE 2009 [5], [6], [7]	ED	3842	3564	3946	3929	4216	4190	4320	4268	4040	4060	3829	3956
		TSS ED	1787	1522	1556	1095	690	354	478	786	1385	1615	1836	1821
		SC	0.4651	0.4271	0.3944	0.2787	0.1637	0.0846	0.1106	0.1843	0.3428	0.3978	0.4795	0.4604
	CTE 2013 [9], [10], [11]	ED	4156	3856	4269	4251	4561	4533	4673	4617	4370	4392	4142	4280
		TSS ED	1980	1688	1726	1217	769	396	533	875	1537	1791	2034	2018
		SC	0.4764	0.4377	0.4043	0.2863	0.1686	0.0874	0.1141	0.1896	0.3517	0.4078	0.4911	0.4716

**Table 7:** Domestic hot water energy demand (ED), in kWh; domestic hot water energy demand met by the thermal solar system (TSS ED), in kWh; and solar contribution (SC) according to commune, regulation and month in solar climate zone F.

Commune	Regulation	Parameter	Jan	Feb	Mar	Apr	May	Jun	Jul	Aug	Sep	Oct	Nov	Dec
Natales	NCL [2], [3], [4]	ED	3631	3332	3689	3655	3874	3844	3982	3943	3759	3806	3608	3602
		TSS ED	1390	1215	1301	919	681	377	458	736	1142	1298	1321	1360
		SC	0.3829	0.3644	0.3527	0.2515	0.1757	0.0980	0.1151	0.1866	0.3038	0.3410	0.3661	0.3777
	CTE 2009 [5], [6], [7]	ED	3873	3555	3935	3899	4133	4100	4247	4205	4009	4060	3849	3842
		TSS ED	1682	1470	1576	1116	825	453	553	893	1386	1574	1599	1645
		SC	0.4342	0.4137	0.4004	0.2861	0.1997	0.1104	0.1302	0.2124	0.3458	0.3876	0.4156	0.4283
	CTE 2013 [9], [10], [11]	ED	4190	3845	4257	4218	4471	4435	4594	4550	4338	4392	4164	4156
		TSS ED	1850	1618	1734	1229	911	502	612	985	1526	1732	1759	1810
		SC	0.4415	0.4207	0.4073	0.2915	0.2038	0.1131	0.1332	0.2166	0.3518	0.3943	0.4226	0.4355
Punta Arenas	NCL [2], [3], [4]	ED	3612	3324	3680	3646	3884	3863	3991	3952	3749	3787	3589	3631
		TSS ED	1421	1173	1228	835	528	243	333	561	1059	1200	1426	1414
		SC	0.3935	0.3528	0.3336	0.2289	0.1358	0.0629	0.0835	0.1420	0.2823	0.3169	0.3973	0.3894
	CTE 2009 [5], [6], [7]	ED	3852	3545	3925	3889	4143	4120	4257	4216	3999	4039	3829	3873
		TSS ED	1851	1532	1605	1095	689	308	429	735	1390	1573	1859	1843
		SC	0.4805	0.4321	0.4090	0.2817	0.1664	0.0748	0.1008	0.1743	0.3476	0.3893	0.4856	0.4758
	CTE 2013 [9], [10], [11]	ED	4168	3835	4246	4207	4482	4457	4606	4561	4327	4370	4142	4190
		TSS ED	2022	1674	1755	1199	757	341	474	807	1521	1719	2031	2013
		SC	0.4852	0.4366	0.4133	0.2851	0.1690	0.0766	0.1028	0.1769	0.3514	0.3935	0.4903	0.4805

## 2 Experimental design, materials, and methods

The thermal solar systems were evaluated for a multifamily building with fifteen 3-bedroom dwellings and five 4-bedroom dwellings, located in the main Chilean communes, with the aim of evaluating the annual solar contribution according to Chilean regulation NCL [2], [3], [4] and Spanish regulations CTE 2009 [5], [6], [7] and CTE 2013 [9], [10], [11]. Thus, it was necessary to set the corresponding annual minimum solar contributions based on the mean annual global solar radiation. The annual solar contribution was obtained through the f-chart algorithm of the Chilean Ministry of Energy to verify the minimum solar contribution of the thermal solar systems [13]. The domestic hot water demands at 45 °C are 2700 l/day for NCL [2], [3], [4], 2880 l/day for CTE 2009 [5], [6], [7] and 3116 l/day for CTE 2013 [9], [10], [11], and the domestic hot water storage tanks selected are 3000 l for NCL [2], [3], [4] and CTE 2009 [5], [6], [7] and 3500 l for CTE 2013 [9], [10], [11]. Each of the solar collectors had an absorption area of 2.23 m^2^, an optical efficiency of 0.811 and an overall loss factor of 3.653 W/m^2^·K. Northern orientation was chosen for the solar collectors, and the inclination was the whole number equivalent to the nearest multiple of 5, corresponding to the geographical latitude of the site. Shading losses were not considered. In no month of the year did the energy produced by the thermal solar system exceed 110% of the domestic hot water energy demand, nor did it exceed 100% of the energy demand for more than three months. The relationship between the volume of the storage tank and the installed surface of the solar thermal collectors was maintained higher than 50 and lower than 180 l/m^2^. The number of solar collectors, the relation between the accumulator volume and the surface of the solar collectors, the annual solar contribution obtained by the thermal solar system, and the minimum annual solar contribution required in each commune to comply with the different regulations, according to solar climate zone, as well as the mean annual global solar radiation and the latitudes of the selected communes, are presented in Table 1. The monthly domestic hot water energy demands, the monthly domestic hot water energy demands met by the thermal solar system and the monthly solar contributions for each commune and regulation are presented according to solar climate zone in Table 2, Table 3, Table 4, Table 5, Table 6, Table 7.
